# Nurses’ perspectives on the implementation of patient-reported outcomes: a descriptive qualitative study

**DOI:** 10.1186/s12912-025-03847-7

**Published:** 2025-09-26

**Authors:** Yu min Zhang, Yang yang Wang, Yu Liu, Chen chen Zhong, Min Zhou, Li Chen, San lan Guo

**Affiliations:** 1 Department of Nursing, Tongji Hospital, Tongji Medical College, Huazhong University of Science and Technology, No. 1095 Jiefang Avenue, Wuhan, 430030 China; 2 Department of Otolaryngology-Head and Neck Surgery, Tongji Hospital, Tongji Medical College, Huazhong University of Science and Technology, Wuhan, 430030 China

**Keywords:** Patient-reported outcomes, Nurses’ perspective, Qualitative study, Patient-centred care

## Abstract

**Background:**

Patient-reported outcomes (PROs) are crucial for understanding patient needs and improving patient-centred care. Nurses are key facilitators in PROs implementation, offering valuable insights. However, their perspectives and challenges remain underexplored. This study aims to investigate nurses’ perceptions, barriers, and support needs in implementing PROs.

**Methods:**

A descriptive qualitative study was conducted, using semi-structured interviews with 17 nurses from a tertiary public hospital in China. Data were analysed using thematic analysis, with post-hoc mapping of emergent themes to the Consolidated Framework for Implementation Research (CFIR). The study adhered to the Consolidated Criteria for Reporting Qualitative Research (COREQ) checklist for thorough reporting.

**Results:**

Three main themes were identified: (1) Reframing the value of patient-centred care through PROs, including enhancing the quality of nurse–patient communication and advancing the professionalisation of nursing practice; (2) Structural barriers to the implementation of PROs, encompassing the cognitive–practical competency gap and the lack of organisational support mechanisms; and (3) Innovation needs and pathways for a digitally enabled, PROs-oriented transformation in nursing, covering institutional pathways for implementation and facilitating integrative innovation through intelligent technologies. CFIR mapping highlighted how individual knowledge and beliefs, organisational culture, and intervention adaptability shaped both facilitators and barriers.

**Conclusions:**

This study demonstrates that while nurses generally have positive attitudes towards the integration of PROs and acknowledge its potential to enhance communication, care quality, and professional practice, significant multi-level barriers remain. Addressing these challenges requires department-wide training, strong leadership, clear policies, and investment in adaptable digital infrastructure. These system-level strategies can empower nurses to use PROs confidently and consistently, thereby supporting the institutionalisation of patient-centred care in China and similar healthcare contexts.

**Clinical trial number:**

Not applicable.

**Supplementary Information:**

The online version contains supplementary material available at 10.1186/s12912-025-03847-7.

## Introduction

The increasing emphasis on patient-centred care has highlighted the need to understand patients’ subjective experiences, thereby prompting the exploration of patient-reported outcomes (PROs) [1]. PROs are direct reports from patients about any aspect of their health status, without interpretation of their responses by physicians or anyone else [2]. The integration of PROs into clinical practice has the potential to enhance patient satisfaction and the quality of care. By capturing details that may otherwise be overlooked, PROs provide comprehensive information to healthcare professionals, improving communication between patients and providers [3, 4]. In contrast to traditional physician-reported outcomes, physiological metrics, and caregiver-reported outcomes, PROs emphasise the subjective experiences and feelings of patients, offering a more nuanced understanding of their well-being [1, 5]. PROs reflect patients’ internal states and needs, and thus serve as a vital tool for enhancing the quality of healthcare.

PROs are systematically collected through Patient-Reported Outcome Measures (PROMs), which are standardised tools (e.g., questionnaires, scales) designed to capture and quantify patients’ subjective health experiences in a structured and reproducible manner [6]. Professional associations and regulatory bodies have recently issued standardised guidelines to support the implementation of PROs in clinical care [7, 8]. Despite these guidelines and supporting evidence, major challenges remain in implementing PROs in practice [9].

Nurses, comprising around 60% of the global healthcare workforce, play a critical role in monitoring patient experiences, communicating directly, and providing timely interventions [10]. As primary caregivers, nurses are ideally positioned to facilitate the implementation of PROs [11]. Nurse-led supportive care models based on PROs have shown potential in improving symptoms and addressing unmet needs in cancer patients, particularly in those with lung [12], colon [13], and cervical cancer [14]. Furthermore, Pedersen et al. [15] reported that Nurse-led symptom management based on PROs significantly improved quality of life (QoL) and reduced symptom burden in patients with chronic haematological malignancies. Therefore, nurses should take a leading role in implementing and utilising PROs to enhance patient health outcomes, improve care quality, and promote individualised care [16].

The perceptions of those implementing PROs are crucial for planning effective implementation and assessment tools [17]. Studies show that nurses with positive attitudes towards PROs are more likely to use standardised assessment tools and integrate more PROs data into treatment planning [18, 19]. Conversely, negative attitudes hinder PROs engagement, increasing the perceived time burden of collecting, searching, and reviewing PROs data [20, 21]. As PROs become increasingly integrated into clinical nursing practice and research in China [22], understanding nurses’ acceptance of PROs is crucial to realising their full potential. However, most research on PROs implementation has focused on Western clinical settings, with limited attention to healthcare systems like China’s [23]. In China, public hospitals play an important role in the healthcare system, accounting for 92% of hospital admissions and 89% of the total bed capacity [24]. Owing to the unique structure of China’s healthcare system, significant differences in PROs implementation outcomes may differ substantially from those in Western settings.

Exploring these differences is critical for understanding and improving the integration of PROs in China. PROs research in China began relatively recently, with most studies focusing on developing measurement scales [25]. The awareness of healthcare professionals regarding PROs, particularly among nursing staff, has received limited attention in the existing literature. Liu Huan [26] and Huang Yue-shi et al. [27] found that although most healthcare professionals recognised the value of using PROs in clinical practice, many expressed concerns about integrating them into routine practice. Huang Yue-shi et al. [27] also reported discrepancies in the awareness and attitudes between doctors and nurses.

Thus, in this distinct healthcare environment, it is essential to explore nurses’ perspectives from various departments regarding implementation practices, perceived barriers, and support needs. To better understand the complexity of PROs implementation, we have utilised the Consolidated Framework for Implementation Research (CFIR). The CFIR is widely recognised in nursing and public health for integrating constructs from multiple disciplines [28, 29]. It consists of five domains—Intervention Characteristics, Outer Setting, Inner Setting, Characteristics of Individuals, and Process [30]—and offers a flexible, theory-informed structure to examine how contextual, organisational, and individual factors influence PROs implementation. In contrast to other frameworks, such as RE-AIM [31] or iPARIHS [32], CFIR does not require a predefined implementation phase. Its adaptability makes it particularly suitable for China’s emerging PROs application, where adoption is in its early stages.

Accordingly, this study aims to explore nurses’ perceptions, barriers, and support needs in implementing PROs in a large tertiary public hospital in China, interpreting these findings through the CFIR framework. The findings will provide context-specific evidence to inform tailored strategies for PROs adoption, strengthen patient-centred care, and guide policy and practice in comparable settings.

## Methods

### Design

This study employed a qualitative descriptive approach with semi-structured, face-to-face interviews. This method, grounded in empiricism and naturalism, systematically collects first-hand data to describe clinical phenomena and nursing practice patterns [33]. It is particularly appropriate for exploratory research, especially in contexts where practical applications are limited and challenges need to be identified [34].

The study explored the perspectives, obstacles, and needs of Chinese nurses in implementing PROs in clinical settings. Given the context-dependent nature of the phenomenon and the complexity of nurse–patient interactions, a design capable of capturing these dynamics was essential. Qualitative descriptive studies help identify the barriers and needs nurses face when implementing PROs. These insights can inform the development of strategies to enhance patient-centred care. To ensure consistent reporting, the study adhered to the Consolidated Criteria for Reporting Qualitative Research (COREQ) checklist [35].

### Setting and sample

The study was conducted at a tertiary hospital in central China, which has over 7,000 beds and employs approximately 4,000 nurses. The hospital is characterised by a hierarchical organisational culture, typical of Chinese public medical institutions, with a strict three-tier structure (frontline nurses → department head nurses → nursing department). This structure requires multi-level approvals for the implementation of new interventions, such as the integration of PROs. Furthermore, nurses are burdened with a substantial workload that includes non-clinical tasks (e.g., insurance audits, research data collection), which may restrict the time available for integrating PROs into clinical practice.The hospital employs an established electronic health record (EHR) system, which integrates patient demographic data, clinical documentation, and nursing assessment tools. However, the integration of PRO-related functionalities within the EHR remains limited, and the system lacks automated alert mechanisms for PROs-related data. Professional development within the hospital includes mandatory annual training; however, PROs-related content is not part of the core curriculum or promotion criteria. In fact, most of the PROs-related knowledge is only presented in occasional professional lectures during meetings, and the hospital does not offer dedicated training on this subject. Consequently, PROs training is fragmented and not incorporated into a hospital-wide, standardised curriculum.

Purposive sampling [36] and snowball sampling were employed in this study. To ensure a diverse and representative sample, maximum variation sampling was used to include nurses with different ages, professional titles, qualifications, and work experiences. This approach was chosen to capture a wide range of perspectives, which is essential for the depth of qualitative analysis. Inclusion criteria included: (a) possession of a valid nursing licence; (b) actively involved in patient care in hospital wards or outpatient clinics; and (c) voluntarily providing informed consent. Exclusion criteria included nurses who were temporarily unable to perform clinical duties (e.g., due to pregnancy, illness, or extended leave) at the time of recruitment.

To identify potential participants, we collaborated with the head nurse who helped us reach out to nurses actively involved in patient care. Recruitment was initiated through formal invitations sent by the head nurse. We approached nurses through a combination of face-to-face invitations during their shifts and WeChat. All participants were provided with detailed information about the study, including its purpose, the voluntary nature of participation, and their right to withdraw at any point. Each participant signed informed consent before the interview, ensuring ethical compliance and respect for participants’ autonomy.

### Data collection

Face-to-face interviews were conducted between August 2021 and January 2022. The interviews were conducted by the primary and secondary authors, both female master’s degree students. They had systematically studied qualitative research and were well-acquainted with interviewing methods and techniques before the interviews. The participants were unfamiliar with the interviewers. The interview guide was meticulously developed through a comprehensive literature review and in-depth discussions with clinical experts. It was piloted with two clinical nurses to refine the questions for clarity and relevance. Their role was to review and clarify any unclear or ambiguous questions in the outline. They were also responsible for adding any missing information to create the final version of the interview outline, presented in Table 1. The qualitative researcher conducted a single, one-on-one interview with each participant, with no repetition of interviews. Interviews were conducted in private settings, either in the hospital ward’s meeting room or the professor’s office, ensuring a quiet and secluded environment with no other individuals present. Audio recording was conducted after obtaining participants’ consent. For participants who were unfamiliar with the term “PROs”, the interviewer provided a concise, neutral explanation of the concept before starting the formal interview, ensuring a common baseline understanding. Each interview lasted approximately 30–45 min. The audio recordings were transcribed within 48 h after the interview. During and after the interviews, on-site notes were taken. The transcript was subsequently sent to the interviewee for review, and any ambiguities were promptly clarified. Data saturation was achieved when no new themes or subthemes emerged during the final interviews. This was determined by continuously comparing new data to existing themes until additional interviews provided no further insights [37]. Participants also reviewed the transcribed texts and preliminary findings, with no substantive changes suggested.

**Table 1:** Guide for conducting semi-structured interviews

No.	Questions
1	Could you briefly describe your understanding of PROs?
2	What is your perspective on the use of PROs in clinical nursing practice?
3	In your opinion, which factors influence the implementation of PROs?
4	Do you have any additional insights regarding other aspects of PROs?

### Data analysis

Interview data were initially analysed using the thematic analysis method described by Braun and Clarke [38]. The analysis followed six key stages: (1) Familiarisation with the data – repeatedly reading the full transcripts to become immersed in the content and identify initial patterns; (2) Generating initial codes – systematically labelling relevant segments of text based on the research objectives; (3) Searching for themes – grouping similar codes into potential themes through iterative comparison; (4) Reviewing themes – refining themes by merging, splitting, or discarding them to ensure internal coherence and distinctiveness; (5) Defining and naming themes – clearly articulating each theme’s core meaning through concise definitions; (6) Producing the report – synthesising the themes into a coherent analytical narrative, capturing both explicit and underlying meanings. Data collection and analysis were conducted concurrently. The first author and co-authors independently reviewed and manually coded the transcripts, generating initial codes, subcategories, and themes. The research team held iterative discussions to refine the coding framework and resolve discrepancies, thereby reaching consensus on the final categorisation.

To strengthen theoretical grounding, the CFIR was applied during the interpretation stage as a secondary analytical lens. While inductive thematic analysis guided the initial development of themes, CFIR provided a complementary deductive structure to explore contextual factors influencing PROs implementation. Two researchers independently coded the data using the CFIR codebook, with discrepancies resolved by a third independent researcher. Four domains—Characteristics of Individuals, Inner Setting, Intervention Characteristics, and Process—were particularly relevant to this study. The Outer Setting domain was not addressed, as the research focused on internal organisational and individual-level factors within a single hospital context.

To ensure both linguistic and conceptual equivalence, all quotations and thematic labels were translated from Chinese into English using a forward–backward translation process. The translated content was reviewed by bilingual experts familiar with both the clinical and cultural context.

### Rigour

This study rigorously followed the COREQ guidelines [35], ensuring methodological rigour through four key dimensions: credibility, dependability, confirmability, and transferability.

Credibility was enhanced by adopting maximum variation sampling. Participants were recruited from 14 clinical departments, including emergency, oncology, and endocrinology, representing a diverse range of professional titles, educational backgrounds, and clinical experience [39]. All interviews were conducted by researchers formally trained in qualitative methodology, who had no prior relationship with the participants. Interviews were conducted in private, quiet locations and audio-recorded with consent. Verbatim transcriptions were completed within 48 h. Data saturation was reached when no new themes or subthemes emerged during the final interviews. Member checking was performed by sending full transcripts to participants, who reviewed the content line-by-line. Additionally, some participants were invited to comment on a summary of the preliminary findings to ensure alignment with their experiences. No substantive changes were suggested. However, participants were not involved in reviewing codes or theme development. This approach strengthened the accuracy of individual accounts while acknowledging the analytical interpretations were researcher-led. Future studies could involve participants in reviewing codes and themes, further confirming the alignment between the analysis and participants’ perspectives. We applied the CFIR to analyse the barriers and facilitators of PROs implementation. This framework enabled us to map emergent themes within key domains, such as individual characteristics, organisational culture, and intervention attributes, offering a structured approach to contextualising our findings.

Dependability was supported by the use of the thematic analysis method described by Braun and Clarke [38]. The interview guide was developed based on an extensive literature review and iterative discussions with clinical experts. Two clinical nurses piloted the guide to optimise clarity and contextual relevance.

Transcripts were independently coded by the first and second authors. All coding decisions were reviewed and approved by the entire research team. A comprehensive audit trail was maintained throughout the study, including raw audio files, anonymised transcripts, the coding framework, and analytical memos. All materials were securely archived for potential future verification and cross-checking [40].

Confirmability was established through ongoing researcher reflexivity during both data collection and analysis. Field notes and reflective journals were systematically maintained to identify and minimise potential bias. Regular team discussions enabled critical appraisal of emerging interpretations, ensuring the findings authentically represented the participants’ perspectives rather than the researchers’ preconceptions, while being mindful of potential positive response bias [41].We acknowledged the potential for positive response bias, particularly social desirability bias, due to the sensitive nature of PROs discussions. To address this limitation, future studies could incorporate anonymous data collection methods and encourage participants to offer both positive and negative reflections during interviews.Triangulation with additional data sources, such as patient feedback or organisational performance data, could further mitigate response bias and improve the robustness of the findings.

Transferability was enhanced by providing detailed contextual information regarding the study setting and participant characteristics. The study was conducted in a large tertiary public hospital in China with over 7,000 inpatient beds and more than 4,000 nursing staff, covering a wide range of clinical specialities. The hospital’s hierarchical structure and existing technology infrastructure, including the electronic health record system, are key factors influencing the integration of PROs in clinical practice. These elements provide a baseline for understanding how such systems might be applied in similar settings.

All participant quotations and thematic labels were translated using a forward–backward translation process, conducted independently by two bilingual experts with clinical backgrounds. Any discrepancies were resolved by a third linguistic expert, ensuring semantic and conceptual equivalence between the original Chinese data and the English translation [42]. Additionally, findings were presented using thick description, enabling readers to assess the relevance and applicability of the results to other clinical settings.

### Ethical considerations

Ethical approval was obtained from the ethics committee (Approval Number: TJ-IRB20210629). All participants provided informed consent and were informed of their right to withdraw at any time. The data collected during the study were kept strictly confidential and used exclusively for scientific research purposes. Participants’ identities were anonymized and represented by symbols in the report.

## Results

### Characteristics of participants

Seventeen nurses participated in the interviews, with no refusals or withdrawals. All participants were female, with an average age of 34 years (ranging from 27 to 42 years) and an average of 9.88 years of nursing experience (ranging from 5 to 17 years). Demographic details of participants are presented in Table 2.

**Table 2:** General information on interviewees (*n* = 17)

ID	Gender	Age (years)	Education level	Working experience (years)	Job title	Department
N1	Female	39	Bachelor	15	Specialist nurse	Geriatrics
N2	Female	30	Bachelor	5	Staff nurse	Emergency
N3	Female	27	Bachelor	5	Staff nurse	Emergency
N4	Female	28	Bachelor	5	Staff nurse	Oncology
N5	Female	33	Bachelor	10	Staff nurse	ICU
N6	Female	33	Master	10	Staff nurse	Gastrointestinal Surgery
N7	Female	40	Master	15	Specialist nurse	Endocrinology
N8	Female	29	Bachelor	7	Staff nurse	Endocrinology
N9	Female	40	Doctor	15	Nurse manager	Neurology
N10	Female	28	Master	5	Staff nurse	Otolaryngology
N11	Female	28	Bachelor	5	Staff nurse	Liver Surgery
N12	Female	42	Master	17	Nurse manager	Haematology
N13	Female	37	Master	13	Staff nurse	Cardiovascular medicine
N14	Female	36	Bachelor	11	Staff nurse	Ophthalmology
N15	Female	27	Bachelor	5	Staff nurse	Paediatrics
N16	Female	43	Master	15	Nurse manager	Paediatrician
N17	Female	38	Master	10	Specialist nurse	Gynaecology and Obstetrics

### Overview of themes and subthemes

Three overarching themes emerged from the thematic analysis: Reframing the Value of Patient-Centred Care through PROs (primarily reflecting the CFIR domains of Characteristics of Individuals—i.e., knowledge & beliefs, self-efficacy—and Inner Setting—e.g., organisational culture, resource availability), encompassing two subthemes: enhancing the quality of nurse–patient communication and advancing the professionalisation of nursing practice; Structural Barriers to the Implementation of PROs (mapped to the CFIR domains of Characteristics of Individuals and Inner Setting, and incorporating Process constructs such as Executing and Reflecting & Evaluating), with two subthemes: the cognitive–practical competency gap and the lack of organisational support mechanisms; and Innovation Needs and Pathways for a Digitally Enabled, PROs-Oriented Transformation in Nursing (aligned with the CFIR domains of Intervention Characteristics—e.g., adaptability, design quality—and Process—planning, executing, reflecting & evaluating), comprising two subthemes: establishing institutional frameworks for effective implementation and facilitating integrative innovation through intelligent technologies. The data analysis process is summarised in Table 3 and described in-depth below.

**Table 3:** Data analysis process with CFIR mapping

Meaning Units (Excerpts from Quotes)	Code	Sub-themes	Themes	CFIR Domains	CFIR Constructs
“PROs are an excellent way to facilitate communication… especially for student nurses…” (N6)	Improved Communication Framework	Enhancing the Quality of Nurse–Patient Communication	Reframing the Value of Patient-Centred Care through PROs	Characteristics of Individuals	Knowledge & Beliefs
“Sometimes we don’t know what to ask… but patients care about things we haven’t mentioned…” (N4)	Standardised Conversational Pathway	Enhancing the Quality of Nurse–Patient Communication	Reframing the Value of Patient-Centred Care through PROs	Inner Setting	Organisational Culture
“Previously, we focused more on blood pressure… now we also pay attention to patients’ emotional distress” (N8)	Holistic Health Evaluation	Enhancing the Quality of Nurse–Patient Communication	Reframing the Value of Patient-Centred Care through PROs	Characteristics of Individuals	Self-efficacy
“In the past, we primarily relied on professional judgement… now we are more proactive…” (N7)	Collaborative Decision-Making	Enhancing the Quality of Nurse–Patient Communication	Reframing the Value of Patient-Centred Care through PROs	Process	Engaging Key Stakeholders
“We encourage in-bed exercises after surgery… PROs help us identify unmet care needs…” (N6)	Early Risk Identification	Advancing the Professionalisation of Nursing Practice	Reframing the Value of Patient-Centred Care through PROs	Inner Setting	Available Resources
“When a patient reports worsening chest tightness… faster than waiting for ECG confirmation” (N2)	Proactive Emergency Response	Advancing the Professionalisation of Nursing Practice	Reframing the Value of Patient-Centred Care through PROs	Intervention Characteristics	Relative Advantage
“Patients who have undergone tracheotomy… switch to nebulisation to enhance comfort” (N10)	Personalised Care Pathways	Advancing the Professionalisation of Nursing Practice	Reframing the Value of Patient-Centred Care through PROs	Intervention Characteristics	Adaptability
“Previously, we relied on complaints… now PROs promptly identify overlooked problems” (N12)	Patient-Centred Quality Evaluation	Advancing the Professionalisation of Nursing Practice	Reframing the Value of Patient-Centred Care through PROs	Process	Executing
“Assessment tools are not always appropriate for the Chinese cultural context…” (N17)	Cultural Adaptability Challenges	The Cognitive–Practical Competency Gap	Structural Barriers to the Implementation of PROs	Intervention Characteristics	Cultural Sensitivity
“We are familiar with assessment tools… lack understanding of scoring systems” (N13)	Interpretation Difficulties	The Cognitive–Practical Competency Gap	Structural Barriers to the Implementation of PROs	Characteristics of Individuals	Self-efficacy
“The rapid workflow in emergency departments… PROs data becomes burdensome” (N3)	Integration Barriers in High-Pressure Settings	The Lack of Organisational Support Mechanisms	Structural Barriers to the Implementation of PROs	Inner Setting	Workflow Compatibility
“Our department lacks familiarity with PROs… no established protocols” (N10)	Lack of Standardised Guidance	The Lack of Organisational Support Mechanisms	Structural Barriers to the Implementation of PROs	Inner Setting	Readiness for Implementation
“To advance PROs implementation… expert leadership in policy development is required” (N16)	Need for Institutional Coordination	Establishing Institutional Frameworks for Effective Implementation	Innovation Needs and Pathways for a Digitally Enabled, PROs-Oriented Transformation in Nursing	Process	Leadership Engagement
“Clear guidelines are necessary… promoting adoption becomes challenging” (N12)	Unified Procedural Guidelines	Establishing Institutional Frameworks for Effective Implementation	Innovation Needs and Pathways for a Digitally Enabled, PROs-Oriented Transformation in Nursing	Process	Planning
“If a system allowed patients to self-assess… efficiency could be improved” (N7)	Intelligent Platform Integration	Facilitating Integrative Innovation through Intelligent Technologies	Innovation Needs and Pathways for a Digitally Enabled, PROs-Oriented Transformation in Nursing	Intervention Characteristics	Design Quality & Adaptability
“Older adults are more accustomed to voice messages… interface should support voice input” (N1)	Adaptation to User Demographics	Facilitating Integrative Innovation through Intelligent Technologies	Innovation Needs and Pathways for a Digitally Enabled, PROs-Oriented Transformation in Nursing	Intervention Characteristics	Customisation for Users

### Theme 1: reframing the value of patient-centred care through pros

This theme primarily maps to the CFIR domains of Characteristics of Individuals—particularly nurses’ knowledge & beliefs about the intervention (i.e., their understanding, perceived value, and attitudes towards PROs) and self-efficacy (i.e., confidence in their ability to apply PROs effectively)—and aspects of the Inner Setting, such as organisational culture and resource availability.

PROs enhanced nurse–patient communication by providing a standardised, structured framework, improving patient engagement and enabling collaborative decision-making. They contributed to a shift towards a more proactive, patient-centred nursing model. From a CFIR perspective, these changes illustrate how individual-level cognitive factors interact with organisational context to support behaviour change and embed new clinical practices. These developments supported more effective care delivery, early risk identification, and a transparent, feedback-driven process for service improvement.

#### Subtheme 1: enhancing the quality of nurse–patient communication

The integration of PROs significantly improved nurse–patient communication by providing a standardised framework that bridged communication gaps, incorporated patients’ subjective experiences, and encouraged collaborative decision-making. This reflects the CFIR construct of Knowledge & Beliefs about the Intervention, whereby increased familiarity and perceived value of PROs enhanced nurses’ willingness to engage in patient-centred dialogue.

By shifting the focus from purely physiological assessments to the patient’s overall health status, PROs enriched the nursing care process. Communication in nursing is often influenced by differences in nurses’ clinical experience, which can hinder effective information exchange. PROs provided a consistent starting point for dialogue, aligning with the CFIR construct of Self-Efficacy (confidence in one’s ability to perform a specific implementation task), as junior nurses gained confidence in initiating comprehensive conversations:

PROs are an excellent way to facilitate communication… especially for student nurses, as they help to build a bridge for interaction. (N6).

In scenarios where communication prompts were lacking or important topics were unintentionally omitted, PROs offered a clear conversational pathway:

Sometimes we don’t know what to ask… but patients care about things we haven’t mentioned. With PROs, they can express what matters most to them. (N4)

PROs contributed to dismantling the traditional physiologically centred assessment model by integrating patients’ subjective experiences into the nursing process, enabling a more comprehensive evaluation:

Previously, we focused more on blood pressure and blood glucose… now we also pay attention to patients’ emotional distress, which helps us manage their conditions more effectively. (N8)

PROs also enhanced transparency in nursing decision-making, supporting a transition from professionally dominated models to a more collaborative approach. This corresponds to the CFIR construct of Engaging Key Stakeholders—here explicitly extended to include patients as active partners in care planning, rather than only clinical staff:

In the past, we primarily relied on professional judgement… now we are more proactive in listening to patients’ concerns and jointly exploring the possibility of adjusting care plans. (N7)

In summary, this subtheme demonstrates how PROs, when framed through the CFIR domains of Characteristics of Individuals (knowledge & beliefs, self-efficacy) and Inner Setting (organisational culture), can act as a mechanism to enhance both the quality and consistency of nurse–patient communication, while fostering a culture of collaborative, patient-centred care. 

#### Subtheme 2: Advancing the professionalisation of nursing practice

The integration of PROs into clinical practice facilitated a shift from reactive to proactive nursing care, aligning with CFIR domains such as Characteristics of Individuals (Knowledge & Beliefs, Self-Efficacy) and Inner Setting (Organisational Culture, Readiness for Implementation). This transformation promoted early intervention and active patient participation, representing a significant advancement towards a more professional and patient-centred nursing approach. PROs contributed to early risk identification, critical in proactive care, particularly in post-surgical recovery. The Knowledge & Beliefs construct within CFIR highlights how nurses’ understanding of PROs allows them to identify unmet care needs and take timely actions. As noted by a nurse: “We encourage in-bed exercises after surgery to promote recovery, but many patients resist due to pain or lack of confidence. PROs help us identify these unmet care needs and intervene in a timely manner.” (N6)In emergency settings, PROs demonstrated their value by signaling risks earlier than traditional monitoring equipment, which corresponds to the CFIR Process domain (Executing and Reflecting & Evaluating), as nurses used PROs to expedite decision-making processes:“When a patient reports worsening chest tightness, we immediately activate the chest pain emergency protocol—it’s faster than waiting for ECG confirmation.” (N2)As PROs integrated into clinical workflows, nursing practices shifted from standardized protocols to refined, patient-specific care pathways. This corresponds with the CFIR Inner Setting domain, particularly Workflow Compatibility and Leadership Engagement, as organizational culture and readiness for implementation played crucial roles in facilitating such transitions:“In our department, patients who have undergone tracheotomy are routinely provided with humidification via a humidification pump. However, when a patient reports that the condensed water from the device causes coughing or choking, we switch to nebulisation to enhance comfort.” (N10)The integration of PROs also transformed the evaluation of nursing quality. Traditionally, the evaluation of nursing quality relied on complaints or subjective judgment, aligning with the CFIR domain of Characteristics of Individuals (Self-Efficacy) in how nurses perceived their ability to use feedback for quality improvement. Unlike previous methods relying on complaints or subjective judgement, PROs enabled more timely and nuanced identification of patient concerns:“Previously, we relied on complaints or subjective judgement. Now, through PROs, we can promptly identify previously overlooked problems, such as dietary arrangements or the frequency of night-time care.” (N12)In summary, this subtheme illustrates how the integration of PROs, through CFIR domains like Characteristics of Individuals, Inner Setting, and Process, has advanced nursing professionalisation. PROs have improved early risk identification, patient involvement, and fostered a more patient-centred, proactive nursing model, enhancing care delivery and quality.

### Theme 2: structural barriers to the implementation of PROs

Within the CFIR framework, this theme maps to the domains of Characteristics of Individuals—particularly knowledge & beliefs, and self-efficacy—and Inner Setting, while also incorporating Process constructs such as Executing (i.e., carrying out the implementation according to plan) and Reflecting & Evaluating (i.e., assessing progress and outcomes). The findings highlight how deficits in cognitive–practical competence, alongside insufficient organisational infrastructure, constrained the ability of nurses to fully integrate PROs into routine care. These barriers operated both at the individual level, where gaps in understanding or skills hindered effective use, and at the organisational level, where workflow incompatibility, cultural misalignment, and leadership inaction impeded adoption.

#### Subtheme 1: the cognitive–practical competency gap

This subtheme aligns closely with the CFIR domains of Characteristics of Individuals (knowledge & beliefs, self-efficacy) and Intervention Characteristics (cultural sensitivity, adaptability), revealing how limited understanding of PROs’ operational aspects constrained their practical application. Although many nurses expressed familiarity with assessment tools, there was limited competence in interpreting PROs scores, selecting appropriate tools for different patient groups, or adapting content to fit cultural contexts.

We are familiar with assessment tools… but we lack understanding of the scoring systems, which makes it hard to explain results to patients. (N13)

The CFIR construct of Cultural Sensitivity is particularly relevant here, as the absence of localisation limited the relevance of certain PROs items for Chinese patients.

Assessment tools are not always appropriate for the Chinese cultural context, so sometimes patients find questions strange or irrelevant. (N17)

From a Process perspective, deficits in Executing emerged where knowledge gaps translated into inconsistent or incomplete application of PROs in clinical encounters.

In summary, this subtheme shows how bridging the cognitive–practical gap—by enhancing both conceptual understanding and operational skill—would strengthen nurses’ self-efficacy and support more consistent, culturally attuned PROs use.

#### Subtheme 2: the lack of organisational support mechanisms

This subtheme maps primarily to the CFIR domain of Inner Setting, particularly the constructs of Readiness for Implementation (i.e., tangible and intangible indicators of organisational preparedness), Workflow Compatibility, and Leadership Engagement.

In many cases, the absence of standardised guidance and clear protocols hindered integration of PROs into established nursing workflows.

Our department lacks familiarity with PROs… there are no established protocols to follow, so everyone does it differently. (N10)

Workflow incompatibility was most evident in high-pressure settings such as emergency departments, where the fast pace made PROs data entry and interpretation burdensome.

The rapid workflow in emergency departments means PROs data becomes extra workload, and sometimes it’s skipped. (N3)

The CFIR construct of Leadership Engagement emerged as a critical factor—where leadership prioritised PROs integration, nurses reported smoother adoption; where it was absent, uptake lagged.

In summary, this subtheme illustrates how organisational readiness, workflow alignment, and engaged leadership are prerequisites for successful PROs implementation in routine nursing practice.

### Theme 3: innovation needs and pathways for a digitally enabled, PROs-oriented transformation in nursing

Within the CFIR framework, this theme maps to the domains of Intervention Characteristics—particularly Adaptability (i.e., the degree to which an intervention can be modified to fit local needs), Design Quality, and Customisation for Users—and Process constructs such as Planning, Executing, and Reflecting & Evaluating.

The findings reveal a shared recognition among nurses that digital platforms and intelligent technologies are essential to embedding PROs sustainably and at scale. These needs fall into two interlinked pathways: creating supportive institutional frameworks and enabling integrative innovation through technology.

#### Subtheme 1: establishing institutional frameworks for effective implementation

This subtheme aligns closely with the CFIR Process constructs of Planning (developing implementation methods in advance), Leadership Engagement, and Reflecting & Evaluating. Nurses emphasised that without institutionalised protocols, training, and dedicated coordination, PROs implementation would remain fragmented and unsustainable.

Clear guidelines are necessary; otherwise, promoting adoption becomes challenging, especially across different wards. (N12)

Leadership engagement was seen as central to ensuring that PROs were prioritised, resourced, and embedded into broader quality improvement agendas.

To advance PROs implementation, we need expert leadership in policy development and ongoing oversight. (N16)

In summary, this subtheme demonstrates how strategic planning, engaged leadership, and systematic evaluation are mutually reinforcing elements for embedding PROs into routine digital nursing practice.

#### Subtheme 2: facilitating integrative innovation through intelligent technologies

This subtheme maps to the CFIR domain of Intervention Characteristics, especially Adaptability, Design Quality, and Customisation for Users, as well as the Process construct of Executing. Nurses envisioned digital solutions—such as patient self-assessment portals, voice-input interfaces for older adults, and integration with electronic nursing records—to improve efficiency and user experience.

If a system allowed patients to self-assess and upload results, efficiency could be improved, and nurses could focus on interpreting and acting on the data. (N7)

Older adults are more accustomed to voice messages… so the interface should support voice input. (N1)

From a CFIR standpoint, these suggestions illustrate adaptability to local patient demographics, high design quality to reduce workflow friction, and the need for well-managed execution to integrate new tools without disrupting care delivery.

In summary, this subtheme shows that digital innovation, when co-designed with frontline users and tailored to patient populations, can accelerate PROs adoption and enhance the sustainability of patient-centred nursing transformations.

## Discussion

This study used the CFIR as a post-hoc analytical tool to examine nurses’ perceptions of the implementation of PROs in clinical settings. This framework provided a structured theoretical foundation for interpreting the barriers and facilitators identified. The study identified the barriers and needs encountered during PROs implementation, highlighting the interplay between individual competencies, organisational context, and intervention characteristics. The majority of nurses recognised the significant role PROs play in improving communication quality between nurses and patients, as well as in promoting the professional transformation of nursing practice. Specifically, PROs provide nurses with a structured communication framework and standardised entry points, enabling them to efficiently collect comprehensive health data from patients. This approach enhanced interaction between patients and the nursing team and fostered a more collaborative decision-making process.

From a CFIR perspective, these findings align closely with the domain of “Characteristics of Individuals”—particularly the construct of “Knowledge and Beliefs about the Intervention”—as nurses’ understanding and perceived value of PROs shaped their willingness to engage in patient-centred dialogue [43]. These findings align with the results of Basch et al. [44], where 83.9% of nurses reported that PROs significantly improved communication quality and efficiency, and 69.6% believed that PROs enhanced the quality of care. Similarly, CFIR-informed research in oncology nursing has demonstrated that integrating patient-reported data into routine workflows can strengthen stakeholder engagement and co-production of care [45]. Additionally, Schick-Makaroff et al. [46] highlighted that PROs accurately assess patient needs and provide real-time data support for nursing practice, thereby assisting nursing teams in developing personalised care plans. PROs underscore the importance of non-physiological factors, such as emotions and mental health, in overall health assessments, which aligns closely with patient-centred care model [47]. Within the CFIR ‘Process’ domain, the findings align with the construct of ‘Engaging Key Stakeholders’, highlighting the active role of patients as contributors to care planning, as explored in Al Sayah et al.‘s (2025) CFIR-guided analysis of patient-reported outcome measures (PROMs) implementation in Alberta, Canada [48]. Through the application of PROs, the transparency of nursing decisions is enhanced, fostering a shift in nursing practice from a professionally led model to a more collaborative and shared decision-making process [49]. In this process, nurses not only rely on their professional judgement but also become more proactive in listening to patients’ concerns and needs, making patients active co-decision makers in the care process. This active participation not only enhances patients’ involvement in managing their health but also improves the individualisation and precision of nursing services.

Despite the considerable value of the PROs-driven patient-centred care paradigm shift, multiple challenges remain in its practical implementation, which—when examined through the CFIR framework—can be conceptualised as barriers across the domains of “Characteristics of “Characteristics of Individuals,” “Inner Setting,” and “Intervention Characteristics”.These barriers reflect systemic misalignments between individual competencies, organisational support mechanisms, and technological infrastructures, aligning with the CFIR construct of “Readiness for Implementation” within the Inner Setting domain [48]. In this study, such contradictions were evident in gaps in nurses’ awareness and practical skills, inadequate institutional protocols, and fragmented technical systems. In particular, gaps in individual awareness and insufficient practical skills present considerable obstacles, consistent with the findings of Huang Yueshi et al. [27].Unlike studies focusing on specific specialties, such as neurosurgery, this research includes 14 clinical departments and uses maximum variation sampling to capture practice differences.The findings reveal common core barriers to PROs implementation (e.g., cognitive gaps, lack of organisational support) across departments, but with specialty-specific manifestations (e.g., integration difficulties in emergency departments due to high workflow pressure, reliance on parental proxy reporting in paediatrics).Notably, recent qualitative studies on PROs implementation in neurosurgery nursing also highlight specific barriers, such as insufficient understanding and support for integrating PROs into daily clinical practice [16]. These findings support the development of a “universal framework + specialty adaptation” strategy for PROs implementation.Within the CFIR “Characteristics of Individuals” domain, these findings correspond to the constructs of “Knowledge and Beliefs about the Intervention” and “Self-Efficacy”, where limited understanding and negative perceptions of PROs among clinical staff, potentially stemming from insufficient training, undermine their ability and willingness to interpret and apply PROs data effectively [50]. The relatively late introduction of the PROs concept in China has resulted in limited opportunities for clinical nurses to systematically study PROs, leading to difficulties in data interpretation and clinical application [51]. Research indicates that implementing specialised training programmes for PROs can significantly enhance nurses’ theoretical understanding of PROs and facilitate their standardised application in clinical settings [52, 53]. Furthermore, training content should be customised to meet the actual needs of healthcare professionals. Sztankay et al. [54] found that healthcare professionals are more inclined to learn how to interpret PROs data, assess its clinical benefits, and implement regular PROs data evaluations. At the organisational level, barriers align with the CFIR “Inner Setting” constructs of “Available Resources” and “Compatibility,” where heavy workloads, absence of standardised workflows, and lack of technical integration hinder the integration of PROs into daily nursing practice [55]. The lack of supportive systems has led to PROs being perceived as an additional burden rather than an integral part of nursing practice. Moreover, the lack of technical support and feedback mechanisms prevents the establishment of a closed-loop process for quality improvement. This is consistent with the findings of Cheung et al. [56]. Roe et al. [57], in a review of 80 large-scale PROs implementation projects across 15 countries, emphasised that strong policy support is critical for sustainability. From a CFIR-informed perspective, addressing these multi-level barriers requires a dual strategy: strengthening “Access to Knowledge and Information” for individuals and “Leadership Engagement” at the organisational level, coupled with clear operational guidelines and integrated technical platforms [48, 55].

In exploring the clinical needs for the implementation of PROs, the integration of digital technology and localisation emerged as critical facilitators, mapping to the CFIR “Intervention Characteristics” constructs of “Adaptability” and “Design Quality and Packaging”.Digital transformation can enable seamless integration of PROs with existing healthcare information systems through intelligent platforms, thereby enhancing data collection efficiency and reducing the workload of nursing staff. Currently, countries such as France, the United States, and the United Kingdom have established adaptive computer systems for PROs electronic data collection networks [17]. However, CFIR-guided analyses underscore the importance of contextual adaptation, where local cultural norms and system interoperability determine the fit of the intervention [58]. Local adaptation is imperative to address unique challenges in China, such as cultural preferences for voice input among older adults and interoperability with existing hospital systems. Existing PRMOs in China face issues of standardisation, scale variety, and quality [27]. From a CFIR standpoint, these needs highlight the construct of “Trialability”, where iterative piloting of locally adapted PROs tools can reduce adoption risk and enhance user acceptance [45]. Therefore, researchers must not only adopt more rigorous methods for translating and introducing PROMs but also develop systems tailored to local contexts. Such cultural and technological localisation will ensure that PROs are both relevant and effective within specific settings, providing valuable insights for their global promotion and implementation.

This study contributes to implementation science by demonstrating the applicability of the CFIR framework within China’s hierarchical healthcare context. While CFIR is widely used in Western settings, this research reveals key differences in how constructs like Leadership Engagement manifest, where administrative mandates from nursing directors—rather than clinical champions—drive PROs implementation. Additionally, by applying CFIR constructs such as Intervention Adaptability, this study addresses PROs implementation challenges unique to China’s system (e.g., workflow integration, cultural localization), offering practical insights for similar settings.

### Limitations

This study has several limitations that should be considered when interpreting the findings.

Firstly, as a qualitative descriptive study conducted in a single tertiary public hospital in China, the findings are not intended to be generalisable. Instead, the study sought to provide context-specific insights that may be transferable to similar clinical environments, particularly in institutions with comparable organisational structures, technological contexts, and professional cultures.

Secondly, although the interviews were conducted by Master’s-level nursing students who had received formal training in qualitative methods, their limited prior experience with data collection may have influenced the depth, fluidity, and probing during interviews.

Thirdly, face-to-face interviews may have introduced a degree of positive response bias, particularly social desirability bias, whereby participants could consciously or unconsciously emphasise favourable experiences with PROs while underreporting challenges. Although several participants expressed critical perspectives during the interviews, it remains plausible that some unfavourable views were moderated or withheld in the presence of an interviewer—particularly when PROs were framed as an innovative, patient-centred initiative. This potential bias may influence the trustworthiness of the findings by distorting thematic balance towards more positive interpretations of PROs implementation. For instance, if nurses underreported time constraints due to perceived expectations to endorse PROs, the theme “Structural Barriers” (e.g., workload pressure cited by N8) might inadequately reflect operational challenges, potentially misleading resource-allocation plans for PROs implementation. To mitigate such bias, future studies could adopt anonymous data collection methods, combine anonymous surveys quantifying barrier severity with purposeful sampling of critical cases in interviews to capture nuanced dissent, and cross-reference nurses’ accounts with patient feedback and organisational performance data.

Fourthly, while data saturation was achieved, some interviews were relatively short, potentially limiting the richness and nuance of the data. In addition, the sample consisted of 17 nurses out of a population of over 4,000, with voluntary participation, which may have restricted the diversity of perspectives. Although maximum variation sampling was employed to increase departmental diversity, certain specialties were represented by only one participant each, limiting the comprehensive exploration of potential differences in perceptions across clinical settings.Future studies with broader, more balanced samples are recommended to address these gaps.

Fifthly, member checking was conducted at the transcript level, enabling participants to verify the accuracy of their accounts, and some were invited to review a summary of preliminary findings.However, participants were not involved in reviewing the detailed coding structure or final thematic framework, which may limit the confirmability and credibility of the thematic interpretations.

Finally, while CFIR provided a valuable post-hoc analytical lens, it was not integrated into the original study design. Consequently, data collection was not systematically tailored to capture all CFIR constructs, and the Outer Setting domain was only marginally explored, potentially constraining complete alignment between emergent themes and the framework’s conceptual architecture. These contextual characteristics should be considered when assessing the transferability of findings to other healthcare systems.

### Practical implications

Based on the CFIR-based analysis, this study provides targeted, evidence-informed recommendations to optimise the integration of PROs into clinical nursing practice. At the administrative level, nursing and hospital leaders should incorporate PROs workflows directly into existing electronic health record (EHR) systems, thus reducing duplication of documentation and minimising additional workload.

Visible leadership engagement—such as active endorsement from senior nursing managers—can foster organisational readiness, demonstrate institutional commitment, and align departmental priorities with PROs implementation objectives.Appointing dedicated PROs coordinators or champions within each department could ensure standardised data collection, facilitate timely follow-up, and offer consistent oversight. This approach would not only strengthen communication between frontline staff and IT teams but also promote staff engagement and enable real-time resolution of emerging issues.

At the policy level, incorporating PROs-related indicators into hospital quality metrics and performance appraisal frameworks would incentivise consistent and sustained adoption. Allocating targeted funding for digital infrastructure—particularly adaptive platforms supporting culturally tailored features, such as voice input for older adults—can address both technological and cultural barriers to uptake. Furthermore, incorporating PROs training into mandatory continuing professional development programmes would ensure consistent competency across departments, thus improving feasibility, acceptability, and long-term sustainability.

## Conclusions

This study provides an in-depth understanding of nurses’ perceptions regarding the implementation of PROs in clinical practice. While nurses expressed strong support for PROs integration and acknowledged its potential to enhance communication, care quality, and professional practice, multiple barriers were identified. These included insufficient knowledge and skills, absence of standardised procedures, limited organisational support, and fragmented technological infrastructure. To support the effective and sustainable implementation of PROs, it is imperative to strengthen institutional mechanisms. This includes the development of structured training programmes, formal policy frameworks, and robust digital infrastructure aligned with local clinical and cultural contexts. The integration of intelligent technologies—such as electronic health records and adaptive data collection tools—can reduce documentation burden, enhance workflow efficiency, and improve data quality. The findings underscore the necessity of system-level strategies that empower nurses to utilise PROs confidently and consistently, thereby promoting the broader uptake of patient-centred care across healthcare settings in China and similar contexts.

Future research should prioritise cross-departmental investigations to capture discipline-specific challenges and facilitate tailored implementation strategies. Longitudinal and mixed-methods studies are also warranted to evaluate the long-term impact of PROs integration on patient-centred outcomes, including symptom management, care satisfaction, treatment adherence, and health-related quality of life.

## Electronic Supplementary Material

Below is the link to the electronic supplementary material.

Supplementary Material 1

Supplementary Material 2

## Data Availability

The data supporting the findings of this study are available from the corresponding author upon reasonable request. All materials, including interview transcripts and analysis codes, are securely stored and can be shared in compliance with institutional and ethical guidelines.

## Abbreviations

PROs: Patient-Reported Outcomes
COREQ: Consolidated Criteria for Reporting Qualitative Research
PROMs: Patient-Reported Outcome Measures
ICU: Intensive Care Unit
CFIR: Consolidated Framework for Implementation Research
EHR: Electronic Health Record
QoL: Quality of Life
